# The role of sex and ovarian hormones in hippocampal damage and cognitive deficits induced by chronic exposure to hypobaric hypoxia

**DOI:** 10.3389/fnins.2022.953417

**Published:** 2022-08-08

**Authors:** Dongyong Zhu, Mengdi Zhang, Bo He, Yixuan Wan, Lei Wang, Fabao Gao

**Affiliations:** Department of Radiology, West China Hospital of Sichuan University, Chengdu, China

**Keywords:** animal model, cognitive and behavioral dysfunction, hippocampal damage, chronic hypobaric hypoxia, sex differences

## Abstract

**Purpose:**

This study aims to investigate the role of sex and ovarian hormones in hippocampal damage and cognitive deficits and behavioral dysfunction in rats induced by chronic exposure to hypobaric hypoxia.

**Methods:**

Six-week-old male and female SD rats were housed for 3 months either in a real altitude (4,250 m) environment as the model of chronic hypobaric-hypoxia (CHH) or in a plain as controls. The animal behavioral and hippocampal neurons at subcellular, molecular, and ultrastructural levels were characterized after CHH exposure.

**Results:**

After 3 months of CHH exposure, (1) male CHH rats’ serum testosterone level was lower than male controls’ whereas female CHH rats’ serum estradiol level was higher than female controls’; (2) Morris water maze test finds that male rats showed more learning and spatial memory deficits than female rats; (3) male rats showed more severe hippocampal damage, hippocampal inflammation, oxidative stress and decreased hippocampal integrity (neurogenesis and dendritic spine density) than female rats; (4) Western blot analysis shows that, compared with the male control group, in male CHH group’s hippocampus, expression of nNOS, HO-1, and Bax protein increased whereas that of Bcl-2 protein decreased; (5) Expression of PON2 protein in male rats (CHH and controls) was lower than female rats (CHH and controls). In addition, CHH exposure decreased the expression of PON2 protein in both male and female rats; (6) qPCR analysis reveals that CHH exposure reduced the gene expression of *N*-methyl-D-aspartate receptor NR2A and NR2B subunits in male rats’ hippocampus. In addition, compared with the sham CHH group, the expression level of PON2 protein decreased in the OVX-CHH group’s hippocampus whereas oxidative stress, neuroinflammation, and degeneration of hippocampal neurons increased in the OVX-CHH group’s hippocampus.

**Conclusion:**

After CHH exposure, male rats were significantly more likely than female rats to develop hippocampal damage, hippocampal neuroinflammation, and cognitive decline and deficits, suggesting that sex and ovarian hormones were significantly involved in regulating the rats’ susceptibility to CHH exposure-induced hippocampal damage.

## Introduction

Among the numerous people living in high-altitude areas for a long time all over the world, about 12 million people live in the Qinghai-Tibet Plateau (West, 2017). In recent years, the migration of lowland people to high-altitude areas has become an increasingly common activity, including students, hikers, leisure climbers, miners, and skiers. In high-altitude areas, lower oxygen partial pressure may reduce the oxygen content in the arterial blood lead to low availability of oxygen, and finally result in tissue hypoxia (Yu et al., 2016; Chauhan et al., 2019). This pathophysiological condition caused by the decreased pressure at high altitude is called “hypobaric hypoxia,” which is the most significant climatic characteristic of a plateau environment. Studies have revealed that long-term exposure to a hypobaric-hypoxia environment may cause changes in motor behavior, attention deficit, memory loss, and cognitive decline (Cramer et al., 2015; Snyder et al., 2018), etc.

Cognitive deficits induced by chronic exposure to hypobaric-hypoxia are mostly maladaptive responses associated with oxidative stress and inflammation (Dheer et al., 2018; Cramer et al., 2019), blood-brain barrier (BBB) dysfunction (Winter et al., 2016), and hippocampal neurodegeneration (Sharma et al., 2017). Oxidative stress and inflammation, as the foremost contributing factors to the development of hypobaric- hypoxia-mediated neuronal damage, may eventually affect cognitive function (Lin et al., 2013; Malairaman et al., 2014).

Paraoxonase 2 (PON2) ≈ 43 kDa, as a widely expressed intracellular enzyme (absent in the serum), belongs to a gene family composed of PON1, PON2, and PON3 (Sulaiman et al., 2019). PON2, anti-inflammatory, and antioxidant in the brain and other tissues (Aguirre-Vidal et al., 2020; Blackburn et al., 2021), is indispensable for maintaining the normal tissue structure. For example, deficiency in PON2 may result in increased susceptibility to oxidative stress and neuroinflammation (Furlong et al., 2016), and even aggravate cardiac remodeling upon cardiac insults (Li et al., 2018). PON2 expression is usually much higher (by about three times in the brain) in female mice than in male mice, which is probably related to the balance of this enzyme regulated by estrogens (Levy et al., 2019).

It has been reported that women and female animals cope better than men and male animals with exposure to decreased oxygen partial pressure at high altitudes (Joseph et al., 2000). Women are usually physiologically protected against hypobaric-hypoxia insults until they reach menopause (Joseph et al., 2002). After menopause, they begin to be susceptible to several hypoxia-associated syndromes at high altitudes such as decreased respiratory function, hypoxemia, excessive erythrocytosis, and chronic mountain sickness (León-Velarde et al., 1997, 2001). This suggests that sex and sex hormones do make a difference in the changes in disease manifestation under chronic hypoxic stress (Soliz et al., 2009). However, even so, there has been a scant amount of studies on sex differences in susceptibility to hippocampal damage induced by chronic exposure to high altitude hypoxic environments.

Given that postmenopausal women have a higher level of oxidative stress and that estradiol is neuroprotective by suppressing oxidative stress (Sánchez-Rodríguez et al., 2012; Xiao et al., 2021), we hypothesize that male animals are probably more vulnerable to hippocampal damage than females when exposed to chronic hypobaric-hypoxia (CHH). To validate this hypothesis, by establishing a hypobaric-hypoxia model composed of young female and male rats in a real plateau environment, we investigated sex differences in hypobaric-hypoxia-mediated hippocampal damage and cognitive function deficits in them. By analyzing and comparing the cognitive behavior of female and male rats, the levels of hormones, oxidative stress parameters, inflammatory factors in the serum, and morphological changes, and by measuring the content and expression of proteins and genes in the rats’ hippocampus, we investigated the sex differences between CHH model rats to provide experimental and theoretical evidence for clarification of the mechanism underlying sex differences in CHH and its potential for therapeutic intervention.

## Materials and methods

### Ethical approval

This study was approved by the Experimental Animal Ethics Committee of West China Hospital, Sichuan University, Chengdu, China. All the experiments were carried out in compliance with the relevant ethical guidelines, regulations and protocols.

### Animals and experimental design

A total of 104 6-week-old male and female Sprague-Dawley rats weighing 160–170 g (Dossy Experimental Animals Co., Ltd., Chengdu, China) were used in the present study. Male (*n* = 52) and female (*n* = 52) rats were randomized into either the chronic hypobaric-hypoxia (CHH) group or the control group. The latter was reared in Chengdu, China, at an altitude of 500 m above sea level (a.s.l.) for 3 months, while the CHH group was transported from Chengdu, China to Yushu, a city in the Qinghai-Tibet Plateau of China at an altitude of 4,250 m a.s.l. and also reared there for 3 months. The CHH rats were transported by a special experimental animal transport vehicle equipped with a constant temperature system that kept the in-vehicle temperature at 21–24°C constantly. All the animals had access to standard rodent chow and tap water ad libitum in all cages (with six rats in each cage), with an environmental temperature of 22–24°C and humidity of 50–60%. Three months later, the CHH group was transported back to Chengdu from the laboratory animal room at Yushu in the Qinghai- Tibet Plateau.

**FIGURE 1:**
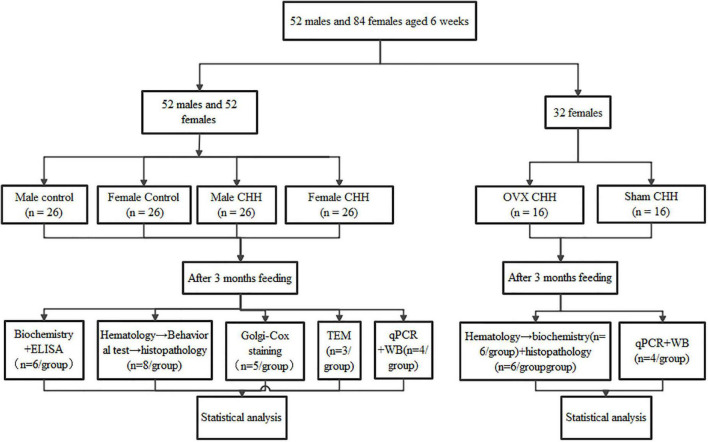
Flow chart of the experiment.

5-bromodeoxyuridine (BrdU) was used as an exogenous proliferation marker. In the S phase of cell division, the marker was integrated into a genetic material. A total of 32 rats in the CHH and control groups were randomly selected (*n* = 8 of each sex per group). From the first day of 2 weeks before the end of 3 months of CHH exposure, the selected 32 rats were intraperitoneally injected once a day with BrdU (Sigma Aldrich, B9285, St. Louis, MO, United States) solution prepared with normal saline at a dose of 100 mg/kg for 7 consecutive days (Figure 1). Two weeks after the final BrdU administration, rats were sacrificed and perfused.

### Analysis of hematological parameters

After 3 months, blood samples from the CHH and control groups were collected from the tail vein under isoflurane anesthesia (*n* = 8 of each sex per group randomly chosen). For routine blood analysis, the coccygeal vein blood (1 ml, with EDTA) was collected with a Mindray automatic hematology analyzer (BC-2800vet, Shenzhen, China). The analysis parameters included white blood corpuscles (WBC), red blood corpuscles (RBC), hemoglobin (HGB), hematocrit (HCT), mean cell volume (MCV), mean corpuscular hemoglobin (MCH), mean corpuscular hemoglobin concentration (MCHC), platelets (PLT), and mean platelet volume (MPV). The analysis was carried out with the Auto Hematology Analyzer (Mindray BC-2800Vet, Shenzhen, China).

### Biochemistry and enzyme-linked immunosorbent assay

#### Preparation of serum and hippocampal tissue samples

Six rats of each sex were randomly selected from each group and were then deeply anesthetized with isoflurane and eventually decapitated. The blood was collected at the bifurcation of the abdominal aorta with a disposable blood collection needle and a coagulation-promoting tube (3 ml). The blood samples were centrifuged at 4°C (3,000 rpm/min) for 10 min to separate the plasma from the whole blood. The supernatant was taken, with the samples with severe hemolysis discarded, and then frozen at –80°C and stored for subsequent analysis. The intact hippocampal tissues were immediately collected into labeled tubes and stored at –80°C until analysis.

#### Detection of estradiol levels in the serum

Serum concentrations of estradiol (cat. no.: E-EL-0152c) and testosterone (E-EL-0155c) were tested using enzyme-linked immunosorbent assay (ELISA) by rat commercial kits (Neobioscience Technology, Shenzhen, China) according to the manufacturer’s instructions.

#### Enzyme-linked immunosorbent assay detection of proinflammatory cytokine levels

Inflammatory markers in the hippocampus were assayed by ELISA, using commercial assay kits. The concentration of IL-1β (cat. no.: ERC007), IL-6 (ERC003), and TNF-α (ERC102a) levels were assessed by using rat ELISA kits (Neobioscience Technology, Shenzhen, China).

#### Biochemical detection of oxidative stress parameters in the hippocampus

According to the manufacturer’s instructions, we measured the levels of superoxide dismutase (SOD) (cat. no.: KTB1030), malondialdehyde (MDA) (KTB1050), glutathione peroxidase (GSH-Px) (KTB0640), glutathione (GSH) (KTB1600), and catalase (CAT) (KTB1040) in the hippocampus with commercial assay kits (Abbkine Scientific Co., Ltd., CA, United States). The total protein concentration of the supernatant was detected by the BCA kit.

##### Lipid peroxidation

The final product malondialdehyde (MDA) was determined by spectrophotometry, as described by Utley et al. (1967). Each MDA molecule reacted with two thiobarbituric acids (TBA) molecules to form a colored MDA-TBA complex, which can be quantified under a 531 nm spectrophotometer. In short, 750 μl 20% TCA and 750 μl 0.67% TBA were added to 250 μl supernatant. The samples were incubated in a water bath at 85°C for 45 min and then centrifuged at 2,000 rpm at room temperature for 5 min. About 200 μl of the supernatant was taken, and the absorbance was determined with a spectrophotometer at 532 nm, using an μQuant spectrofluorophotometer (Bio-Tek, Winooski, VT, United States). The molar extinction coefficient on the MDA-TBA complex (i.e., 1.56 × 105 cm^2^mmol^–1^) was used to estimate the formed MDA, and the resultant value was expressed in nanomoles/mg protein.

##### Glutathione

The reduced glutathione (GSH) levels were measured from the crude homogenate by fluorescence method according to the method of Hissin and Hilf (1976). In short, an equal volume of 10% metaphosphoric acid was added to the 250 μl crude homogenate, and then centrifuged at 10,000 rpm at 4°C for 30 min. The supernatant thus obtained was used to evaluate GSH content by fluorescence spectrometry under 412 nm excitation emission.

##### Glutathione peroxidase, catalase, and superoxide dismutase enzyme activities

With the assay kit of GSH-Px, CAT, and SOD, the enzyme activities of GSH-Px, CAT, and SOD were measured at 340, 540, and 450 nm by spectrophotometry, respectively.

### Behavioral assessment

#### Open field test

Several experimental studies have reported that simulated CHH exposure contributes to emotional behavioral changes, such as anxiety-like behavior (Nguyen et al., 2021; Chauhan et al., 2022). The hippocampus has also been heavily implicated in depression and anxiety (Bannerman et al., 2004; Campbell et al., 2004; Mehta et al., 2013). Open field test (OFT) is a classical behavioral test to assess the locomotor activity and anxiety-like behaviors of rodents. OFT apparatus used in this study consisted of a black plastic square (110 cm × 110 cm × 25 cm) with a video tracking system hanging over the open-field arena that could automatically record the activity of the animal in the arena. Light bulbs (25 W) from overhead illuminated the center of the arena. At the beginning of OFT, each rat was placed in the middle of the open-field arena and its locomotor behavior was recorded for 5 min. OFT behavior was analyzed according to the total distance covered and time spent by the rat in the central area.

#### Morris water maze

Morris water maze (MWM) test is a hippocampal-dependent assessment of spatial learning by rodents (*n* = 8/per group) (Wang et al., 2016; Zhan et al., 2019). In the training trials, the rats were released into the water facing the wall of the pool from one of the four randomly assigned release points (N, W, S, and E, respectively), and allowed to swim until they landed on the platform. Once the rats landed on the platform, they were allowed to rest on it for 10 s. The time the rats took from entering the water to finding the platform and climbing onto the platform was recorded as the escape latency. If a rat failed to climb onto the platform within 60 s, the time was recorded as 60 s. After that, the rats were guided to rest on the platform for 15 s. Each animal was trained on the platform at a fixed location four times a day for 5 consecutive days. In the probe trials, 24 h after the last training concluded, the platform was removed, and the animals were released into the water from the opposite quadrant to track their movement trajectory for 60 s. The swimming time, distance, and speed were recorded and analyzed by the SMART digital video tracking system (Version 3.0, Panlab, Harvard, Holliston, MA, United States).

### Magnetic resonance imaging acquisition and data analysis

Imaging experiments were conducted after behavior assessment (*n* = 8/group). All MRI examinations were performed in a 7.0 T magnetic resonance imaging system (BioSpec 70/30; Bruker, Karlsruhe, Germany). For the rat studies, a 72-mm open birdcage quadrature volume resonator was utilized for excitation, and a four-channel (2 × 2) phased array coil was used as a receiver. Anesthesia was initially induced with 2.5% isoflurane and maintained by 1–1.5% isoflurane (2 L/min oxygen flow) during MRI scanning. The rat was placed in the prone position with the head immobilized with a toothed rod and two ear pins. A respiratory rhythm sensor was installed under the abdomen to observe respiration and monitored at 50-80 breaths per minute. Also, the rats were kept on an animal heating pad connected with hot circulating water, which was maintained at 37 ± 1.0°C during scans to prevent the body temperature from dropping.

For the voxel-based morphometry, high-resolution anatomical MRI data were captured using a T2-weighed turbo-rapid acquisition with relaxation enhancement (RARE) sequence. These T2-weighted images (T2WI) were acquired using a 2D RARE sequence with the following parameters: repetition time (TR) = 11,400 ms, echo time (TE) = 48 ms, number of excitations (NEX) = 6, slice thickness = 0.35 mm, slice gap = 0 mm, field of view (FOV) = 35 mm × 35 mm, in-plane resolution of 0.137 mm^2^ × 0.137 mm^2^ (matrix size = 256 × 256), and total scan time of approximately 38 min. A prior method (Zhu et al., 2022) for VBM data processing was adopted. The statistical threshold used the family-wise error (FWE) setting with a *p*-value of 0.05. If corrected results were not able to survive FWE, the uncorrected method was selected at *p* < 0.0001, cluster size = 100.

### Histopathological examination

Following behavior assessment, the brains of all the rats were collected and underwent morphologic studies. The animals were euthanized with an overdose of isoflurane and perfused transcardially with 250 ml of ice-cold saline, followed by 300 ml of 4% paraformaldehyde (PFA) in phosphate-buffered saline (PBS, 0.1 mol/L, pH = 7.4) for 30 min at 12 ml⋅min^–1^. After the perfusion, the brains were extracted and fixed in 40 ml of 4% paraformaldehyde. After sufficient post-fixation, the brains were embedded in paraffin. A total of 4-μm-thick coronal sections were consecutively taken from about bregma –3.11 mm by sled microtome.

#### Fluoro Jade-B staining

For the neurodegeneration study, the brain sections of the rats were stained with Fluoro Jade-B (FJB) dye, which is a fluorescent substance that binds sensitively and specifically to degenerating neurons. The staining was performed by referring to Scallet et al. (2004). After the staining, degenerating neurons were counted under a fluorescence microscope (Nikon Eclipse C1, Tokyo, Japan). Images of CA1, CA3, and ventral dentate gyrus (DG) granule cells (upper lobe) were obtained using a 40× magnification. FJB-positive neurons were manually counted in the hippocampal sub-regions of interest (CA1, CA3, and DG) per rat section (*n* = 6 rats/group) and each area in 3–5 sections using “Image-Pro Plus 6.0” software (MediaCybernetics, Silver Spring, MD, United States). The slides were independently assessed by two observers blinded to the status of the rats, and the average results of their assessment were regarded as the final data.

#### Double immunofluorescence staining

After the paraffinized brain slides were dewaxed and rehydrated, the sections were incubated with one of the following two primary antibodies, anti-NeuN (1:500, Servicebio)/anti-Iba1 (1:1000, Servicebio) and anti-NeuN (1:500, Servicebio)/anti-BrdU antibody (1:100, Servicebio) in blocking serum at 4°C overnight. The following day, the sections were incubated with secondary antibodies consisting of Alexa Fluor 488 goat antimouse and Cy3 goat antirabbit for 1 h in the dark at room temperature. The cell nucleus was counterstained with DAPI for 10 min in the dark. The sections were covered with anti-fade mounting medium. Each of the above was washed three times for 5 min with PBS. Iba1-labeled cells were counted in the hippocampal subregions of interest (CA1, CA3, and DG) under an immunofluorescence microscope (Nikon Eclipse C1, Tokyo, Japan). For double labeling of BrdU and NeuN, the images were observed by a confocal microscope (Nikon A1R, Nikon, Japan), and co-labeled BrdU^+^/NeuN^+^ cells were counted and quantified by Image-Pro Plus 6.0 software (Media Cybernetics, Silver Spring, MD, United States). About 3–5 sections per area per rat (*n* = 8 rats/group) were analyzed, and the mean number of positive cells in the hippocampus was counted in each image.

### Golgi-Cox staining and image analysis

The dendrites and the spines in the hippocampus are critical carriers for learning and memory function. To evaluate the effect of plateau hypoxic exposure on them, the neural morphology in the hippocampus was assessed. A modified Golgi-Cox staining method was used for the analysis of hippocampal dendrites and spines as previously described (Aguilar-Hernández et al., 2020). After being deeply anesthetized and perfused intracardially with 0.9% saline solution, the brain tissues were removed and cut with a blade into 2–3 mm thick slabs from sections about bregma –3.1 mm for the hippocampus and processed with an FD Rapid GolgiStain^®^ kit (FD Neuro Technologies, Ellicott City, MD, United States). The brain was impregnated in a Golgi-Cox mixture of solution (provided by the kit producer) for 2 weeks in the dark at 37°C and then transferred into a protectant solution prior to paraffin embedding for 48 h according to the manufacturer’s instructions. The brains were coronally sectioned into slabs of 100 μm using an oscillating microtome (Cryotome E, Thermo, Waltham, MA, United States) and the slabs were collected on 5% gelatin-coated microscope slides. The slides were then stained in concentrated ammonia, fixed with sodium thiosulfate, dehydrated in gradient ethanol, and cleared in xylene until mounting.

The hippocampal neurons in CA1 and CA3 regions [*N* = 5 rats per group and *n* = 4–5 neurons per region in each hemisphere (8–10 neurons per animal) selected randomly per rat] were imaged at 200-fold magnification, and the spines were imaged at 1000-fold magnification with a Nikon wide-field microscope (Eclipse Ci-L). The total dendritic lengths were quantified by the Neuron J Plugin for ImageJ/Fiji software (National Institutes of Health, Bethesda, MD, United States). Sholl analysis with the ImageJ program was used to analyze the dendritic complexity by counting the number of intersections across dendrites with an overlaid concentric sphere at 10-μm intervals from the cell soma (Semple et al., 2017). The spines were counted manually using ImageJ. For dendritic spines density analysis, the number of spines along the dendritic segment (30–90 μm range length), which was selected from the second or third proximal branch of apical dendrites on each fully stained neuron, were counted, and mean spine density values were obtained spines/10 μm. At least two dendritic segments ranging from 30 to 90 μm in length were analyzed per hippocampal neuron, and 8–10 neurons in each hippocampal region (CA1, CA3, and DG subregion) of individual rats (*n* = 5 rats per group) were examined for dendritic spine density.

### Western blot analysis

Protein detection was performed by Western blot. The rat hippocampus was fully homogenized in a glass homogenizer and incubated with 400 μl RIPA lysis buffer (including PMSF and protease inhibitor) for 30 min on ice. Then the lysates were centrifuged at 13,000 rpm for 10 min at 4°C, and the protein supernatants were used for protein quantification with enhanced bicinchoninic acid (BCA) Assay Kit (Thermo Fisher Scientific, Waltham, MA, United States). SDS-PAGE electrophoresis was performed with equal amounts of protein (∼30 μg per sample). After being transferred to PVDF membranes, the blots were blocked with non-fat dried milk solution 5% (w/v) containing 5% BSA at 37°C for 2 h. Afterward, they were incubated with primary antibodies (1:1000, Huabio, Hangzhou, China) and GAPDH (1:10000, Huabio, Hangzhou, China) overnight at 4°C. After being washed with TBST, the HRP binding secondary antibody was incubated at room temperature for 2 h. The protein bands were captured using the ECL detection system (Model 680, Bio-Rad Laboratories, Inc., Hercules, CA, United States) and analyzed with NIH ImageJ software (National Institutes of Health, Bethesda, MD, United States).

### RNA isolation and quantitative real-time polymerase chain reaction

Total RNA was extracted from the rats’ hippocampal tissues with Trizol reagent (Invitrogen, Carlsbad, CA, United States). cDNA was synthesized and quantitative real-time PCR amplification reactions were performed. The RNA solution of 2 μg sample was added into the PCR tube, and 2 μl of AccuRTReaction Mix (4×) was added, carefully supplemented to 8 μl with enzyme-free water, and placed at room temperature for 5 min. About 2 μl volume of AccuRT Reaction Stopper (5×) was added and mixed gently. About 4 μl 5× All-in-One RT Master Mix and 6 μl enzyme-free water were added in turn to the above reaction system, total volume: 20 μl. PCR was performed according to the following procedures. First, it was maintained at 25°C for 10 min, then maintained at 42°C for 15 min, and at 85°C for 5 min. After the above steps, the reaction system was cooled on ice. Quantitative PCR took 0.2 ml PCR tubes and prepared three tubes for each reverse transcription product according to the following reaction system. 10.0 μl volume of 2× qPCR Mix; 1.2 μl volume of 7.5 μM gene primer; 2.0 μl volume of retrotranscript; 6.8 μl volume of double distilled water. PCR amplification conditions were as follows: (1) Pre-denaturation: at 95°C, pre-deforming for 10 min; (2) Thermal cycle (repeat 40 times): heating at 95°C for 15 s followed by annealing for 1 min, lasting for 40 cycles at 60°C; (3) Melting curve: 60→95°C, rising by 0.3°C every 15 s. PCR of every sample was repeated three times, and the data were normalized to GAPDH expression and expressed as a fold change compared to the control by the 2^–ΔΔCt^ method. The sequences of primers used in this study are listed in Table 1.

**TABLE 1:** Primer sequences for qRT-PCR assays.

Gene	Sequence (5′-3′)	Length (bp)	Tm(°C)
GAPDH	Forward: ACAGCAACAGGGTGGTGGAC Reverse: TTTGAGGGTGCAGCGAACTT	226	60
NR2A	Forward: AGCCCCCTTCGTCATCGTAGA Reverse: ACCCCTTGCAGCACTCTTCAC	186	60
NR2B	Forward: TGAGACTGAGGAGCAAGAGGATGAC Reverse: GCTTCTGGCACGGGACTGTATTC	128	60
nNOS	Forward: AATGGTGGAGGTGCTGGAGGAG	112	60
	Reverse: GTCTGGAGAGGAGCTGATGGAGTAG		
HO-1	Forward: CAGGTGTCCAGGGAAGGCTTTAAG	96	60
	Reverse: TGGGTTCTGCTTGTTTCGCTCTATC		

### Transmission electron microscope

Ultrastructural changes in the hippocampus (CA1, CA3, and DG) were observed using a transmission electron microscope (TEM) (JEOL Ltd., Tokyo, Japan). The rats (*n* = 3 rats/group) were deeply anesthetized using isoflurane as abovementioned, decapitated and their brains were perfused consecutively with 2.5% glutaraldehyde perfusate via the heart. Their hippocampi were carefully dissected into slabs of about 1 mm thick, immediately fixed in 3% glutaraldehyde (buffered at pH 7.4) for 18–20 h, and postfixed in 1% osmium tetroxide for 2 h at 48°C. Following the dehydration, the hippocampi were embedded in acetone and epoxy resin (Epon 812), and the ultrathin sections of 50 nm thick were fixed on copper. After being stained with 1% uranyl acetate and lead citrate at room temperature, the ultrastructure of the hippocampal and cortical neurons was observed using a JEM-1400FLASH TEM (JEOL Ltd., Tokyo, Japan).

### Ovariectomy

Ovariectomy (OVX) was performed to verify whether ovarian hormones were involved in CHH exposure-induced hippocampal neuronal degeneration in female rats. Another 32 6-week-old female rats were randomized into two groups, the OVX-CHH group and the sham CHH group (*n* = 16 animals/group). Ovariectomy was performed under anesthesia induced by 1% ketamine (110 mg/kg, i.p.), following standard procedures (Balapattabi et al., 2021). In the sham CHH group, the ovaries were not removed while only the same amount of adipose tissue around the ovaries was removed, and other operation steps were the same. Penicillin was injected intraperitoneally for 3 days to prevent infection. After 7 days of postoperative recovery, the rats were transported to the above-mentioned high-altitude areas for observation and feeding under the above conditions. After 3 months of exposure to high altitude hypoxia, the serum and hippocampal tissues were collected for ELISA, histological, and protein expression analysis as described above. The uterus was removed and weighed according to the previous procedure (Chang et al., 2021) to confirm a complete ovariectomy.

### Statistical analysis

The statistical software Prism Version 8.0 software (GraphPad Software, La Jolla, CA, United States) and SPSS 26.0 (IBM Corporation, Armonk, NY, United States) were used for analysis and graphics. When conforming to a normal distribution, the data were expressed as the mean ± standard deviation ($\bar{x}$ SD). The Shapiro–Wilk method was used to test the normal distribution of data, and the Levene method was used to test the homogeneity variance of data. When the data were unsatisfactory according to Mauchly’s test of sphericity, the Greenhouse–Geisser correction was used. An independent sample *t*-test or Mann–Whitney non-parametric test was used between the two groups. The analysis of variance (ANOVA) of the factorial design was used for the comparative analysis among multiple groups. The group (CHH group/control group) and sex (male/female) were used as the independent variables between the subjects, and the training days (days 1, 2, 3, and 4) in the training trials of the Morris water maze were used as the variables within the subjects (repeated measurement). When there is an interaction between factors, simple effects were further analyzed. The Sidak method was selected for *post hoc* pairwise comparison to evaluate the potential differences of various indexes between the control and CHH groups of each sex. *p* < 0.05 was statistically significant.

## Results

### Chronic hypobaric-hypoxia-exposure effect on hematological parameters

Hematological parameters of the rats are shown in Table 2, which shows that compared with the male control group, the weight of male CHH rats increased significantly (*p* < 0.05) whereas there was no significant difference between the female control group and the female CHH group in body weight (*p* > 0.05). The table also shows that CHH exposure significantly increased HGB, HCT, MCV, and MPV in both male and female rats (*p* < 0.05) whereas MCHC decreased significantly (*p* < 0.05) and that CHH exposure significantly increased the RBC of female rats (*p* < 0.05). Albeit RBCs an increasing trend in the male CHH group, the difference was not statistically significant (*p* = 0.088).

**TABLE 2:** Parameters of hematology in male and female rats with or without CHH exposure.

	Male		Female	
	Control-M	CHH-M	Control-F	CHH-F
Weight				
Basic weight (g)	166.6 ± 1.21	164 ± 3.77	162.8 ± 3.44	165 ± 2.61
Weight of 3 months later (g)	379.21 ± 10.77#	411.18 ± 8.38*#	273.57 ± 3.88	274.88 ± 4.06
Hematology analysis				
WBC (million/mm^3^)	4.44 ± 1.61	3.22 ± 2.16	2.57 ± 1.54	2.66 ± 0.21
RBC (million/mm^3^)	8.84 ± 0.57	9.49 ± 0.36	7.43 ± 0.7*#	8.8 ± 0.46
HGB (g/dL)	165.63 ± 7.82	191.38 ± 9.26*#	141.14 ± 13.90*#	172.75 ± 10.95
HCT (%)	50.66 ± 2.37	66.61 ± 4.2*#	43.84 ± 3.95*#	58.88 ± 3.3
MCV (%)	57.46 ± 3.15	70.16 ± 2.26*	59.07 ± 1.58#	67.01 ± 2.85
MCH (pg)	18.80 ± 1.06	20.16 ± 0.64	19.01 ± 0.59	19.67 ± 1.20
MCHC (g/L)	327.38 ± 17.50	287.5 ± 13.41*	322 ± 9.52#	293.75 ± 7.85
PLT (10^9^/L)	840 ± 138.51	688.13 ± 184.29	970.14 ± 185.45	783.88 ± 244.9
MPV (fL)	8.49 0.64	11.54 ± 0.51*	7.83 ± 0.80#	10.75 ± 0.58

Values are means ± SD for 8 animals/group.

WBC, white blood corpuscles; RBC, red blood corpuscles; HGB, hemoglobin; HCT, hematocrit; MCV, mean cell volume; MCH, mean corpuscular hemoglobin; MCHC, mean corpuscular hemoglobin concentration; PLT, platelets; MPV, mean platelet volume.

Data for male and female rats with or without CHH exposure were analyzed using two-way ANOVA. Post hoc analysis indicated: *p < 0.05 vs. male control group; #p < 0.05 vs. female CHH group.

### Effect of chronic hypobaric-hypoxia exposure on serum sex hormone level

Serum sex hormone level obtained by ELISA is shown in Table 3. The serum estradiol level of the female CHH group was significantly higher than the female control group (*p* = 0.048). There was no significant difference in serum estradiol level between the male CHH group and the control group (*p* > 0.05) [main effect of group: *F*(1,20) = 5.23, *p* = 0.033; main effect of sex: *F*(1,20) = 28.76, *p* < 0.001; the interaction: *F*(1,20) = 2.88, *p* = 0.105]. Serum testosterone level in the male CHH group was significantly lower than the male control group (*p* = 0.017) whereas there was no significant difference between the female CHH group and the control group (*p* > 0.05) [main effect of group: *F*(1,20) = 11.52, *p* = 0.003; main effect of sex: *F*(1,20) = 9.04, *p* = 0.007; interaction: *F*(1,20) = 1.68, *p* = 0.21].

**TABLE 3:** Initial and final body weights, sex hormone, inflammatory factors, and oxidative stress parameters in male and female rats with or without CHH exposure.

	Male		Female	
	Control-M	CHH-M	Control-F	CHH-F
Serum hormone level				
Testosterone (ng/ml)	1.12 ± 0.13	0.72 ± 0.02*	0.45 ± 0.04*	0.31 ± 0.07
Estradiol (pg/ml)	0.47 ± 0.07	0.58 ± 0.09#	1.53 ± 0.10#	1.56 ± 0.17
Proinflammatory factor				
IL-1β (pg/mg)	41.94 ± 3.03	63.85 ± 6.80*	56.39 ± 3.58#	67.78 ± 8.38
IL-6 (pg/mg)	678.86 ± 62.13	65.77 ± 2.54*#	45.43 ± 3.10	646.83 ± 60.62
TNF-α (pg/mg)	112.20 ± 10.34	953.19 ± 54.69*	642.98 ± 72.62	138.92 ± 4.88
Oxidative stress parameters				
MDA (nmol/mg)	34.07 ± 2.75	21.43 ± 2.25*	33.90 ± 3.74	24.94 ± 1.63
SOD (U/mg)	1.10 ± 0.06	1.75 ± 0.06#*	0.84 ± 0.07	0.63 ± 0.07
GSH-Px (U/μg)	100.33 ± 3.58	65.71 ± 4.91*	115.64 ± 6.74*	78.92 ± 7.03
GSH (μg/mg)	0.037 ± 0.001	0.024 ± 0.004*	0.028 ± 0.001	0.030 ± 0.002
CAT (nmol/min/mg)	0.10 ± 0.01	0.14 ± 0.01*	0.12 ± 0.01#	0.13 ± 0.01

Values are means ± SD for 8 animals/group.

T, testosterone; E2, estradiol; IL-1β, interleukin-one beta; TNF-α, tumor necrosis factor-alpha; SOD, superoxide dismutase; MDA, malondialdehyde; GPx, glutathione peroxidase; GSH, glutathione; CAT, catalase.

Data for male and female rats with or without CHH exposure were analyzed using two-way ANOVA. Post hoc analysis indicated: *p < 0.05 vs. male control group; #p < 0.05 vs. female CHH group.

### Levels of proinflammatory cytokines in the hippocampus

Hippocampal ELISA results (Table 3) reveal that the levels of inflammatory markers IL-1β (*p* = 0.015), IL-6 (*p* = 0.027), and TNF-α (*p* < 0.001) in the male CHH group were significantly higher than the male control group. IL-1β level in the hippocampus of the female CHH group was significantly higher than the female control group (*p* = 0.025). There were no significant differences in IL-6 (*p* > 0.999) and TNF-α (*p* = 0.315) in the hippocampus between the CHH group and the control group. The above statistical results indicate that the male CHH rats showed higher levels of inflammatory factors than the control group.

### Parameters of oxidative stress in the hippocampus

The biochemical results of the hippocampal tissue show that compared with the male control group, the content of MDA in the male CHH group increased significantly (*p* = 0.004) whereas the content of SOD (*p* = 0.004) and the activities of antioxidant enzymes such as GSH (*p* = 0.017), CAT (*p* = 0.002) and GSH-Px (*p* = 0.002) decreased significantly. CAT enzyme activity in the female CHH group was significantly lower than in the female control group (*p* = 0.001). Compared with the female control group, the differences in the contents of MDA (*p* = 0.734) and SOD (*p* = 0.1), the activities of GSH (*p* = 0.122) and GSH-Px (*p* = 0.947) in the female CHH group were not statistically significant between the groups. The above statistical results indicate that oxidative stress levels were more significant in the male but not in the female CHH rats when compared with the corresponding control group (Table 3).

### Chronic hypobaric-hypoxia exposure-induced anxiety-like behaviors in male and female rats

**FIGURE 2:**
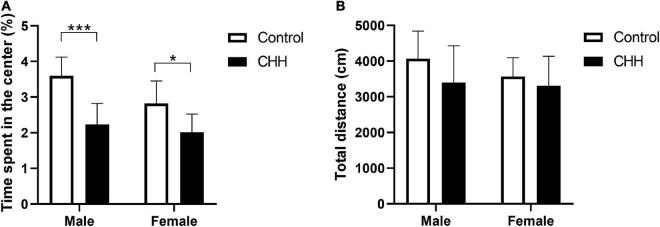
Chronic hypobaric-hypoxia (CHH) exposure induced anxiety-like behavior in rats. **(A)** Percentage of time spent in the center area in the OFT for male and female rats; **(B)** total distance. CHH, chronic hypobaric hypoxia. Values are means ± SD for eight animals/group. Data for male and female rats, with or without CHH exposure, were analyzed using two-way ANOVA. *Post hoc* analysis indicated: *p < 0.05, **p < 0.01, ***p < 0.001.

In the OFT, the percentage of time spent in the central arena was affected by the main effect of group [*F*(1,28) = 29.39, *p* < 0.001] and main effect of sex [*F*(1,28) = 6.17, *p* = 0.019] but no interaction between group and sex [*F*(1,28) = 1.95, *p* = 0.174]. *Post hoc* analysis shows that the percentage of time spent in the central arena was significantly reduced in both male and female CHH groups compared with the corresponding male and female control groups (*p* < 0.001 and *p* = 0.048, respectively; Figure 2A), indicating both CHH-exposed male and female rats exhibited anxiety-like behavior. There was no significant difference in total distance between CHH and control groups [Group: *F*(1,28) = 2.63, *p* = 0.116; sex: *F*(1,28) = 1.04, *p* = 0.316; the interaction *F*(1,28) = 0.52; *p* = 0.478] (Figure 2B). The above results imply that CHH exposure had effects on the anxiety-like behavior of the male and female rats but did not affect their spontaneous activity.

### Chronic hypobaric-hypoxia exposure-reduced spatial learning and declarative memory in male rats

**FIGURE 3:**
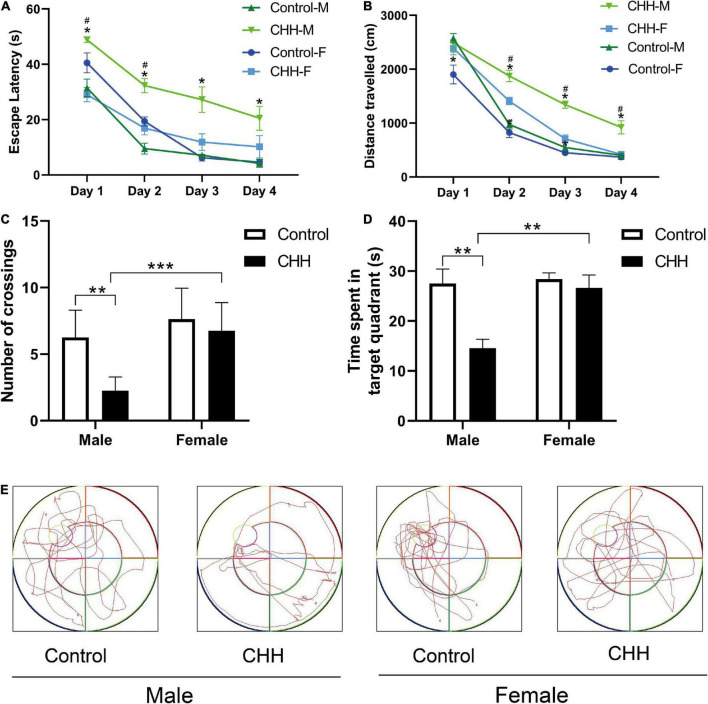
Chronic hypobaric-hypoxia exposure impairs spatial learning and memory in rats. **(A)** Escape latency given in seconds; **(B)** path length given in centimeters; **(C)** the crossing times over the location of the platform; **(D)** time spent in target quadrant; **(E)** representation of the pattern in the probe trial. CHH: Chronic hypobaric hypoxia. Values are means ± SD for eight animals/group. Data for male and female rats, with or without CHH exposure, were analyzed using two-way ANOVA. *Post hoc* analysis indicated: *p < 0.05, **p < 0.01, ***p < 0.001.

In the training trials, the three-way repeated measures ANOVA method was adopted, and the effect of the within-subject factor on training days was significant [*F*(1.77,49.51) = 122.9, *p* < 0.001]. The main effect of the group (with and without CHH exposure) was significant [*F*(1,28) = 22.5, *p* < 0.001], indicating that CHH exposure increased the escape latency of the CHH rats compared with the control group. The main effect of sex was significant [*F*(1,28) = 7.43, *p* = 0.011], indicating that the escape latency of the male rats was longer than that of the female rats (Table 4). Sidak’s *post hoc* analysis shows that the escape latency of CHH-exposed male rats was longer than the male control rats on days 1–4 (*p* < 0.05); however, on days 1–4, the escape latency was not significantly different between CHH-exposed female rats and the control female rats (*p* > 0.05) (Figure 3A). The swimming distance before finding the hidden platform within the training days of rats in each group decreased with the increase in the number of training days (using the method of three-way repeated measures ANOVA, the effect of within-subject factors in training days was significant [*F*(3,112) = 287.6, *p* < 0.001]. The interaction between training days and the group was significant [*F*(3,84) = 7.53, *p* < 0.001], indicating that CHH exposure’s effect was significant (Table 4). Sidak’s *post hoc* analysis shows that the swimming distance of CHH-exposed male rats was longer than the control male rats on days 2–4 (*p* < 0.05); on days 1–4, there was no significant difference in swimming distance between CHH-exposed female rats and the control female rats (*p* > 0.05) (Figure 3B). Overall, after CHH-exposure, the escape latency and swimming distance before finding the hidden platform by male rats rather than by female rats were longer than the control group, indicating that the spatial learning ability of the male rats is more vulnerable to CHH exposure than their female counterpart.

**TABLE 4:** Detailed statistics of three-way ANOVA in the training trials of MWM test.

	Three-way RM ANOVA (Group × sex × time)
	
Escape latency	Time: *F*_1.77,49.51_ = 122.9, *p* < 0.001
	Group: *F*_1,28_ = 22.5, *p* < 0.001
	Sex: *F*_1,28_ = 7.43, *p* = 0.011
	Group × sex: *F*_1,28_ = 25.87, *p* < 0.001
	Time × group: *F*_3,84_ = 3.83, *p* = 0.013
	Time × sex: *F*_3,84_ = 0.97, *p* = 0.041
	Group × sex × time: *F*_3,84_ = 3.72, *p* = 0.014
Swimming distance	Time: *F*_3,112_ = 287.6, *p* < 0.001
	Group: *F*_1,28_ = 63.42, *p* < 0.001
	Sex: *F*_1,28_ = 36.27, *p* < 0.001
	Group × sex: *F*_1,28_ = 2.92, *p* = 0.099
	Time × group: *F*_3,84_ = 7.53, *p* < 0.001
	Time × sex: *F*_3,84_ = 0.39, *p* = 0.760
	Group × sex × time: *F*_3,84_ = 8.32, *p* < 0.001

In the probe trials (Figures 3C–E), the male CHH group had the least number of crossings across the original platform [two-way repeated measures ANOVA, main effect of group was significant *F*(1,28) = 12.51, *p* = 0.001, indicating that CHH exposure affected the number of crossings across the platform; main effect of sex was significant *F*(1,28) = 18.17, *p* < 0.001; sex and group interaction was significant *F*(1,28) = 5.14, *p* = 0.031]. The percentage of time spent in the target quadrant decreased by the male CHH rats [two-way repeated measures ANOVA, the main effect of group was significant *F*(1,28) = 10.73, *p* = 0.003; the main effect of sex was significant *F*(1,28) = 8.30, *p* = 0.008; the interaction between sex and group was significant *F*(1,28) = 6.21, *p* = 0.019]. In the interaction between sex differences, the male rats showed more deficits in spatial memory ability, indicating that CHH exposure varied in its effects on cognitive function between the two sexes.

### Voxel-based morphometry

**FIGURE 4:**
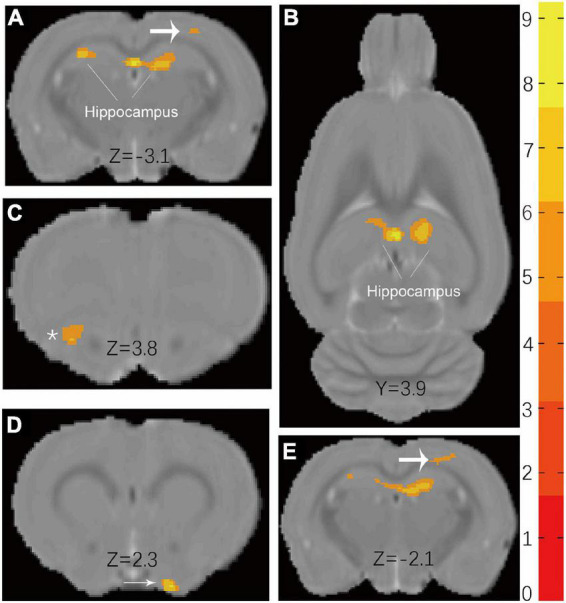
Results **(A,B)** of VBM detect atrophy in hippocampus of male CHH rats in coronal slice and axial slice, compared to male control rats. In addition, coarse arrow **(A,E)**, * **(C)**, and thin arrow **(D)** also show a significant atrophic effect in the primary somatosensory cortex, olfactory bulb, and basal forebrain region, respectively. (The male CHH group, *n* = 8; male control group, *n* = 8; uncorrected *p* < 0.0001). Color bar units refer to *t*-scores.

Voxel-based morphometry reveals that the t-maps for gray matter volume (GMV) were generated after the total brain volumes were controlled for. All macro-structural alterations did not survive *FWE* corrections but when displayed with an uncorrected *p* < 0.0001. The macro-structural alterations showed several regional volume reductions prominently in the hippocampus (Figure 4). In other gray matter areas, the primary somatosensory cortex, the olfactory bulb, and the basal forebrain region were affected. Table 5 shows the significant differences with the cluster size exceeding 100 voxels (2-sample *t*-tests, *p* < 0.0001, uncorrected) between male groups (CHH *vs*. control). There was no significant difference between the female groups (CHH *vs.* control) (data not shown).

**TABLE 5:** The peak of the local maxima within each significant cluster (*p* < 0.0001, uncorrected) showed significant regional gray matter volume decreases in the plateau rats compared with those in the plain rats.

	Coordinates (*x, y, z*)	Peak intensity	*Z* score	Voxels in cluster
Hippocampus	(0.15, 3.89, –3.11)	9.549	4.71	993
Primary somatosensory cortex	(2.4, 5.23, –2.06)	6.6363	4.02	102
Olfactory bulb	(–2.7, 0.29, 3.79)	6.5065	3.98	108
Basal forebrain region	(2.1, –2.42, 2.29)	8.0032	4.38	105

The atlas coordinates of the peaks are relative to bregma in the medial–lateral (x), superior–inferior (y), and anterior–posterior (z) directions (mm).

### Fluoro Jade-B straining

**FIGURE 5:**
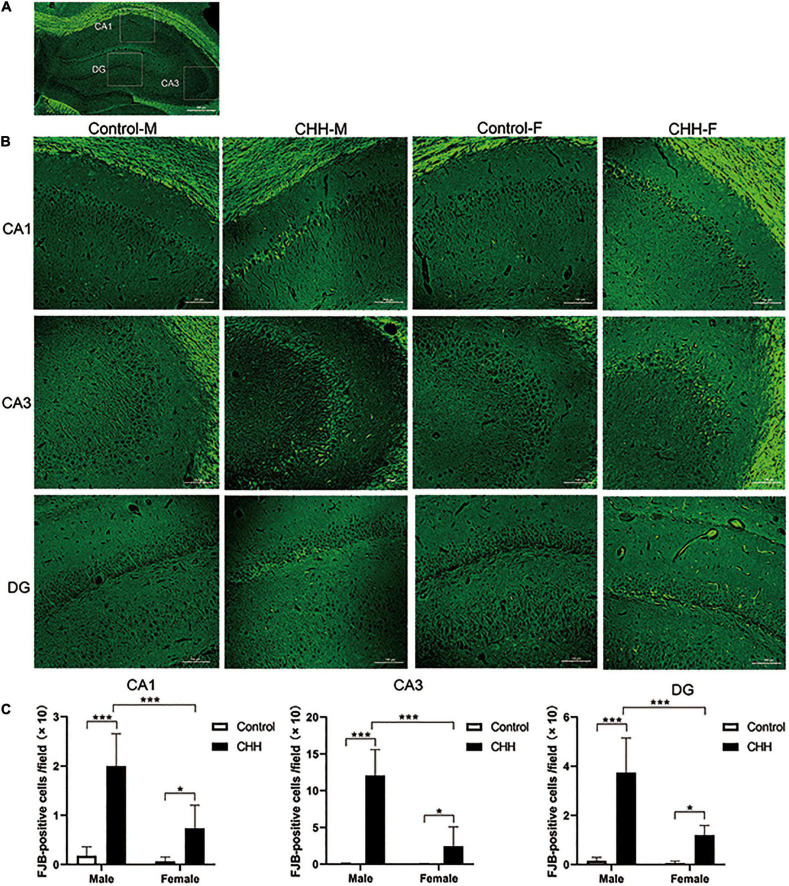
The FJB staining in the hippocampus for degenerative neurons. **(A)** Representative scheme of the CA1, CA3, and DG subregions considered in the analysis. **(B)** Representative images viewed at 200 magnification for FJB staining, scale bar: 100 mm. **(C)** The statistical analysis of FJB positive cells in the hippocampal CA1, CA3, and DG regions is shown. Values are means ± SD for six animals/group. Data for male and female rats, with or without CHH exposure, were analyzed using two-way ANOVA. *Post hoc* analysis indicated: *p < 0.05, **p < 0.01, ***p < 0.001.

Representative scheme of the CA1, CA3, and DG subregions considered in the analysis is shown in Figure 5A. Fluoro Jade-B staining shows that CHH exposure induced neuronal degeneration in the male and female rats (Figure 5B). The two-way ANOVA analysis shows that the main effect of group (with and without CHH exposure) was significant in different hippocampal subregions [CA1: *F*(1,28) = 72.13, *p* < 0.001; CA3: *F*(1,28) = 84.68, *p* < 0.001; DG: *F*(1,28) = 81.94, *p* < 0.001]. The main effect of sex (male and female) was significant in different hippocampal subregions [CA1: *F*(1,28) = 21.82, *p* < 0.001; CA3: *F*(1,28) = 38.19, *p* < 0.001; DG: *F*(1,28) = 25.29, *p* < 0.001], and the interaction between the two sexes was significant in different hippocampal regions [CA1: *F*(1,28) = 15.26, *p* < 0.001]. CA3: *F*(1,28) = 37.6; *p* < 0.001; DG: *F*(1,28) = 22.03, *p* < 0.001. Compared with the control group, the number of FJB positive cells in the CHH groups (male and female) was higher than that in the control group (male and female) (*p* < 0.05). Moreover, the male CHH group showed more FJB-positive cells than the female CHH group (*p* < 0.05) (Figure 5C). However, there was no significant difference in FJB positive cell count between the male and female control groups. Thus, we may say that CHH exposure aggravated neuronal degeneration in both male and female rats and resulted in more severe neuronal degeneration in males than in females.

### Iba-1 immunofluorescence

**FIGURE 6:**
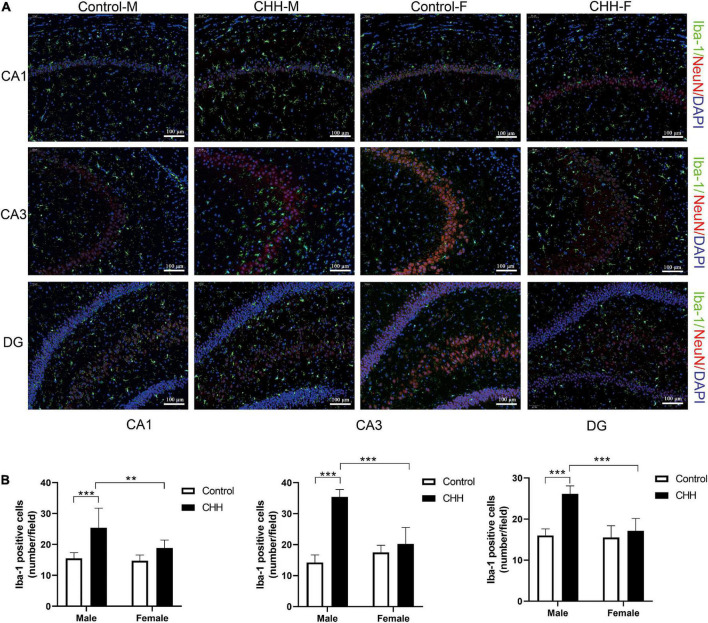
The effects of CHH exposure on the number of microglia. **(A)** Representative images of Iba-1 immunofluorescence staining in the hippocampal CA1, CA3, and DG regions, scale bar: 100 μm. **(B)** The statistical analysis of Iba-1 positive cells in the hippocampal CA1, CA3, and DG regions is shown. Values are means ± SD for six animals/group. Data for male and female rats, with or without CHH exposure, were analyzed using two-way ANOVA. *Post hoc* analysis indicated: *p < 0.05, **p < 0.01, ***p < 0.001.

To verify the increased hippocampal neuroinflammation, the number of Iba-1 positive cells in the hippocampus was counted by immunofluorescence staining (Figure 6A). By two-way ANOVA analysis, the results show that the main effect of group was significant [CA1: *F*(1,28) = 29.02, *p* < 0.001; CA3: *F*(1,28) = 100.7, *p* < 0.001; DG: *F*(1,28) = 46.09, *p* < 0.001]. The main effect of sex was significant [CA1: *F*(1,28) = 7.78, *p* = 0.009; CA3: *F*(1,28) = 24.92, *p* < 0.001; DG: *F*(1,28) = 30.13, *p* < 0.001]. There was a significant interaction between group and sex [CA1: *F*(1,28) = 4.9, *p* = 0.035; CA3: *F*(1,28) = 59.67, *p* < 0.001; DG: *F*(1,28) = 24.12, *p* < 0.001]. Compared with the control group, the number of Iba-1 positive cells in the male CHH group’s hippocampus significantly increased (*p* < 0.001). The number of microglia in the male CHH group was significantly higher than the female CHH group (*p* < 0.01), whereas no significant difference was observed in the number of microglia between the female CHH group and the female control group (*p* > 0.05) and there was no significant difference between the male and female control groups (*p* > 0.05) (Figure 6B).

### Neurogenesis

**FIGURE 7:**
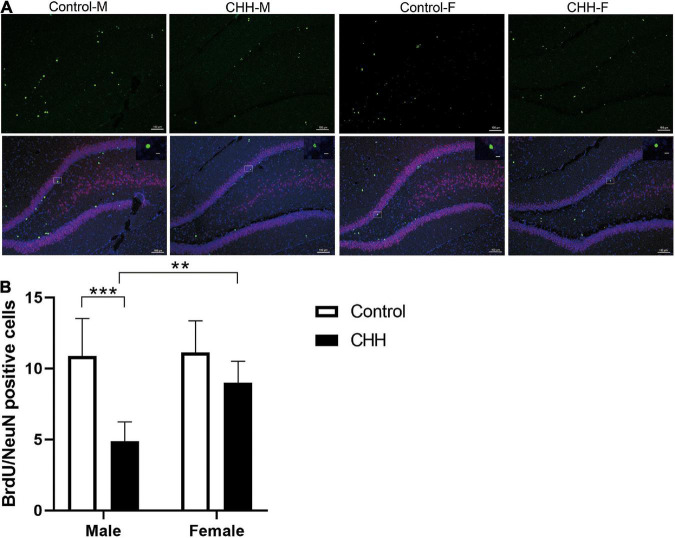
Effects of CHH on neurogenesis in DG region of female and male rats. **(A)** Representative NeuN (green) and BrdU (red) staining in the dentate gyrus of brain sections. **(B)** Quantification of BrdU^+^/NeuN^+^ cells. Values are means ± SD for six animals/group. Data for male and female rats, with or without CHH exposure, were analyzed using two-way ANOVA. *Post hoc* analysis indicated: *p < 0.05, **p < 0.01, ***p < 0.001.

It has been reported that the expression of mature neuronal marker NeuN in BrdU-retaining cells in the hippocampal subgranular zone represents newly-born neurons (Figure 7A). Compared with the male control group, the BrdU/NeuN double positive cells in the male CHH group’s hippocampus decreased significantly (*p* < 0.05) whereas the difference between the female CHH and the female control groups was not significant (*p* > 0.05) [main effect of group: *F*(1,28) = 32.82, *p* < 0.001; main effect of sex: *F*(1,28) = 9.52, *p* = 0.005; the interaction: *F*(1,28) = 7.47, *p* = 0.011] (Figure 7B).

### Dendritic complexity of hippocampal neurons

**FIGURE 8:**
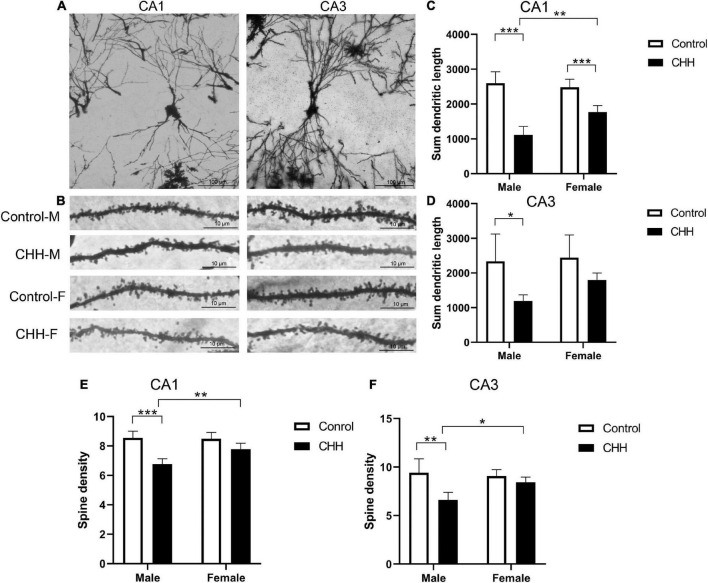
Effects of CHH on dendritic spines of hippocampal neurons in female and male rats. **(A)** Golgi-Cox staining showed dendritic branches in CA1 and CA3 regions, scale bar: 100 μm; **(B)** Golgi-Cox staining showed dendritic spine images in CA1 and CA3 regions, scale bar: 10 μm; **(C,D)** compared with the control group, the total dendritic length in CA1 **(C)** and CA3 **(D)** regions of female rats in CHH group decreased; **(E,F)** compared with the control group, neurons from female CHH rats also showed a decrease in the number of dendritic spines in the CA1 **(E)** and CA3 **(F)** regions. M, male; F, female. Values are means ± SD for five animals/group. Data for male and female rats, with or without CHH exposure, were analyzed using two-way ANOVA. *Post hoc* analysis indicated: *p < 0.05, **p < 0.01, ***p < 0.001.

To further explore sex differences in the effects of CHH exposure on neuronal morphology, we examined dendritic morphology and synaptic ultrastructure (Figures 8A,B). Golgi-Cox staining shows that in CA1 region, the total length of dendrites was affected by group: *F*(1,16) = 93.69, *p* < 0.001; sex: *F*(1,16) = 5.71, *p* = 0.03; the interaction between group and sex: *F*(1,16) = 11.59, *p* = 0.004. *Post hoc* analysis finds that the total length of hippocampal dendrites in the CHH group was significantly shorter than the control male and female rats (*p* < 0.05; Figure 8C). In the CA3 region, the total length of dendrites was not statistically significant long in sex [*F*(1,16) = 2.22, *p* > 0.05; Figure 8D).

Dendritic spine density analysis shows that in CA1 region, dendritic spine density was affected by main effect of group [*F*(1,16) = 44.44, *p* < 0.001] and main effect of sex [*F*(1,16) = 6.51, *p* = 0.021], and the interaction between group and sex [*F*(1,16) = 8.37, *p* = 0.011]. *Post hoc* analysis reveals that, in the CA1 region, the dendritic spine density of hippocampal neurons in the male CHH group was significantly lower than in the male control group (*p* < 0.05; Figure 8E), whereas there was no significant difference between the female CHH group and the female control group (*p* > 0.05). There was no sex difference between CHH and control groups in the dendritic spine density of hippocampal neurons in the CA3 region [*F*(1,16) = 3.22, *p* > 0.05; Figure 8F].

### Measurement of nNOS, HO-1, PON2, Bcl-2, and Bax protein by Western blot in the hippocampus

**FIGURE 9:**
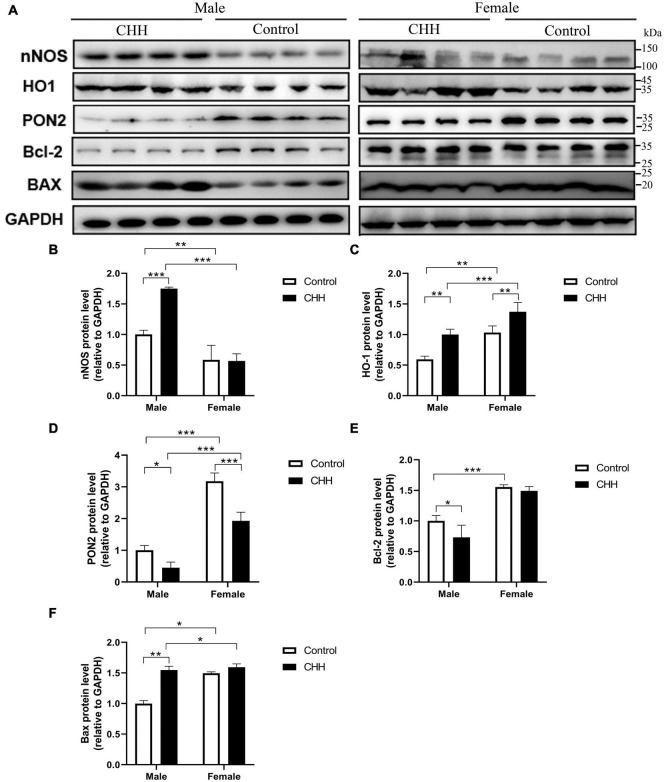
Effects of CHH exposure on the levels of the following signaling proteins from the hippocampus of male and female rats: nNOS, HO-1, PON2, Bcl-2, and Bax. The signaling proteins were measured by immunoblot analysis and were normalized to GAPDH as a reference protein in the hippocampus. Data are reported as fold induction relative to the male control group for male rats. Representative bands are shown on the top panel **(A)**. Representative Western blots of results shown for **(B)** nNOS, **(C)** HO-1, **(D)** PON2, **(E)** Bcl-2, and **(F)** Bax in the hippocampus. Values are means ± SD for four animals/group. Data for male and female rats, with or without CHH exposure, were analyzed using two-way ANOVA. *Post hoc* analysis indicated: *p < 0.05, **p < 0.01, ***p < 0.001.

The expression levels of nNOS, HO-1, PON2, Bcl-2, and Bax proteins in the hippocampus were examined by Western blot (Figure 9A). The expression levels of nNOS, HO-1, PON2, and Bcl-2 proteins in the hippocampus were examined by Western blot. Compared with the male control group, the expression level of nNOS protein in the hippocampus of CHH-exposed male rats increased (*P* < 0.05) whereas there was no significant difference in the expression level of nNOS protein between the female groups (*p* > 0.05), indicating that there was a sex difference. [Group: *F*(1,12) = 28.5, *p* < 0.001; sex *F*(1,12) = 136.2, *p* < 0.001; the interaction: *F*(1,12) = 31.55, *p* < 0.001] (Figure 9B).

Compared with their corresponding control groups, the expression level of HO-1 protein in the hippocampus of the male CHH group and the female CHH group increased significantly (all *p* < 0.05), indicating that CHH exposure raised HO-1 protein expression level in male and female rats [Group: *F*(1,12) = 48.38, *p* < 0.001; sex *F*(1,12) = 56.98, *p* < 0.001; the interaction: *F*(1,12) = 0.37, *p* = 0.556] (Figure 9C).

The expression level of PON2 protein in the hippocampus of female rats (CHH group and control group) was significantly higher than male rats (CHH group and control group) (*p* < 0.05). After 3 months of CHH exposure, the expression level of PON2 protein decreased in the hippocampus of both female and male CHH rats (*p* < 0.05). [Group: *F*(1,12) = 65.91, *p* < 0.001; sex: *F*(1,12) = 271.3, *p* < 0.001; the interaction: *F*(1,12) = 10.03, *p* = 0.008] (Figure 9D).

In male CHH-exposed rats, the hippocampal expression level of Bcl-2 was significantly lower than in the control group (*P* < 0.05) whereas no significant difference was found between the female CHH group and the control group (*p* > 0.05). [Group: *F*(1,12) = 8, *p* = 0.015; sex: *F*(1,12) = 36.64, *p* < 0.001; the interaction: *F*(1,12) = 3.17, *p* = 0.1] (Figure 9E).

In male CHH-exposed rats, the hippocampal expression level of Bax was significantly higher than in the control group (*p* < 0.05) whereas no significant difference was observed between the female CHH group and the control group (*p* > 0.05). [Group: *F*(1,12) = 175.2, *p* < 0.001; sex: *F*(1,12) = 121.3, *p* < 0.001; the interaction: *F*(1,12) = 84.17, *p* < 0.001] (Figure 9F).

### Measurement of nNOS, HO-1, NR2A, and NR2B mRNA expression in the hippocampus

**FIGURE 10:**
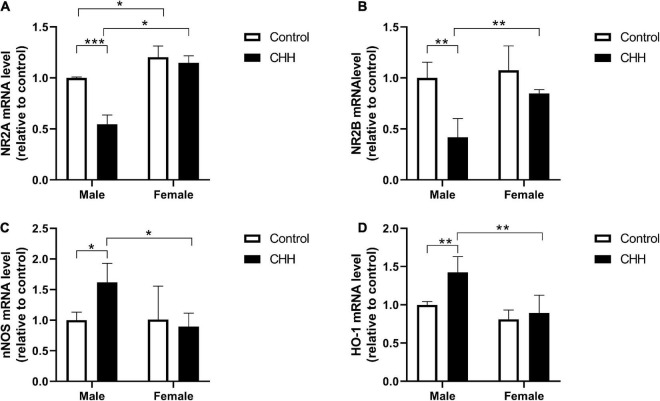
Quantitative analysis of NR2A, NR2B, nNOS, and HO-1 mRNA in the hippocampus of male and female CHH and control rats. CHH exposure reduced the gene expression of *N*-methyl-D-aspartate receptor **(A)** NR2A and **(B)** NR2B subunits and increased the gene expression of **(C)** nNOS and **(D)** HO-1 in male rats’ hippocampus. Values are means ± SD for four animals/group. Data for male and female rats, with or without CHH exposure, were analyzed using two-way ANOVA. *Post hoc* analysis indicated: *p < 0.05, **p < 0.01, ***p < 0.001.

The mRNA expression levels of nNOS, HO-1, NR2A, and NR2B in the hippocampus were evaluated by qPCR analysis. The results show that compared with the male control group, the hippocampal NR2A mRNA level of the male CHH group decreased significantly (*p* < 0.05), but not in female rats (*p* > 0.05) [Group: *F*(1,12) = 40.49, *p* < 0.001; sex: *F*(1, 12) = 101.8, *p* < 0.001; the interaction: *F*(1,12) = 25.35, *p* < 0.001] (Figure 10A).

Compared with the male control group, the hippocampal level of NR2B mRNA of the male CHH group significantly decreased (*p* < 0.05), and the hippocampal level of NR2B of the female rats showed a downward trend. Nevertheless, there was no statistical difference compared with the female control group (*p* > 0.05). [Group: *F*(1,12) = 22.57, *p* < 0.001; sex: *F*(1,12) = 8.88, *p* = 0.012; the interaction: *F*(1,12) = 4.38, *p* = 0.058] (Figure 10B).

Compared with the male control group, the hippocampal level of nNOS mRNA in the male CHH group was significantly higher (*p* < 0.05), but not in the female CHH group (*p* > 0.05) [Group: *F*(1,12) = 2.21, *p* = 0.163; sex: *F*(1,12) = 4.35, *p* = 0.057; the interaction: *F*(1, 12) = 4.75, *p* = 0.05] (Figure 10C). Compared with the male control group, the hippocampal level of HO-1 mRNA in the male CHH group was significantly higher (*p* < 0.05) but not in the female CHH group (*p* > 0.05) [Group: *F*(1,12) = 9.05, *p* = 0.011; sex: *F*(1,12) = 18.38, *p* = 0.001; the interaction: *F*(1,12) = 4.09, *p* = 0.066] (Figure 10D).

### Transmission electron microscope results

**FIGURE 11:**
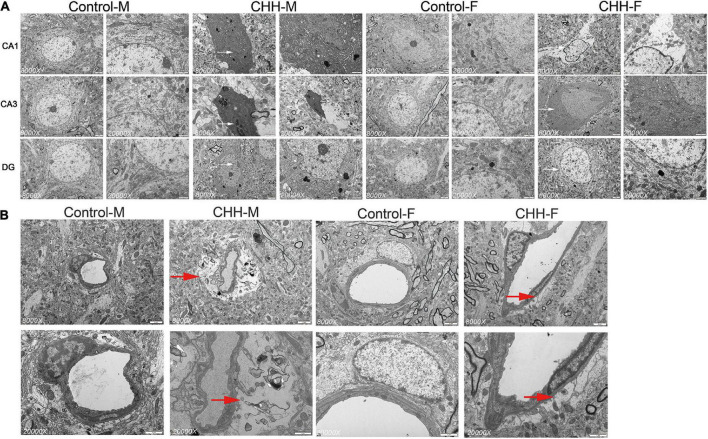
Ultrastructure of hippocampal neurons and blood vessels in female and male CHH groups and control groups **(A)** shows the ultrastructure of neurons in hippocampal CA1, CA3, and DG regions; Early apoptosis or apoptotic signs (white arrow) and swelling of neuronal mitochondria were observed in CHH group; **(B)** It shows the ultrastructure of the vessels in the hippocampal CA3 area. The red arrow in the micrograph shows the widening of the space around the blood vessel (red arrow). **(A)** Scale bar = 2 μm. **(B)** Scale bar = 1 μm.

The hippocampal subregions (CA1, CA3, and DG) were observed at the ultrastructural level. The male CHH group showed severe apoptotic death of neuronal cells (Figure 11A). However, the hippocampus of the female CHH rats showed less neuronal damage and relatively intact cell morphology compared with the male CHH rats. There was no obvious abnormality in the ultrastructure of neurons in the above areas of the male and female control rats. The ultrastructural changes in the hippocampal vessels in the CHH group are shown in Figure 11B. In the male CHH rats, the widened extravascular perivascular space of the hippocampus was observed whereas there was no obvious abnormality in the ultrastructure of hippocampal vessels in the female CHH group and the control group.

### Effects of ovariectomy on oxidative stress and pro-inflammatory cytokines in the hippocampus of female chronic hypobaric-hypoxia-exposed rats

The OVX results show that compared with the sham-CHH group, the weight of female rats in the OVX-CHH group increased significantly (*p* > 0.05). The weight of the uterus of female rats in the OVX-CHH group decreased significantly, and the level of the serum estradiol decreased significantly, which evidences the effectiveness of ovariectomy in this experiment. Compared with the sham-CHH group, there was no significant difference in hematological indexes between the OVX-CHH group and the sham-CHH group (*p* > 0.05). Compared with the sham CHH-group, the proinflammatory cytokines (IL-1β, IL-6, TNF-α) in the hippocampus of female OVX-CHH rats increased significantly (*p* < 0.05). Compared with the sham-CHH group, the content of MDA in the hippocampus of female rats in the OVX-CHH group increased and the activity of antioxidant enzymes (SOD, GSH-Px, CAT) decreased (*p* < 0.05) (see Table 6).

**TABLE 6:** Effects of OVX on body weight, serum sex hormones, hippocampal inflammatory factors, and oxidative stress parameters in female rats exposed to CHH.

	Sham-CHH	OVX-CHH	*P-*value
Weight (*n* = 8/group)			
Basic weight (g)	199 ± 3.12	200 ± 1.24	0.370
Weight after 3 months (g)	276.28 ± 3.16	345.41 ± 5.73	<0.001
Uterine weight (g) (*n* = 8/group)	412.15 ± 18.13	189.34 ± 8.45	<0.001
Hematological analysis (*n* = 8/group)			
WBC (million/mm^3^)	2.46 ± 0.22	3.09 ± 2.03	0.078
RBC (million/mm^3^)	8.79 ± 0.62	9.03 ± 0.72	0.459
HGB (g/dl)	174.45 ± 10.45	179.13 ± 14.81	0.344
HCT (%)	56.48 ± 3.19	61.30 ± 5.09	0.277
MCV (%)	66.01 ± 2.75	67.88 ± 1.80	0.481
MCH (pg)	19.57 ± 1.15	19.81 ± 0.18	0.735
MCHC (g/L)	294.85 ± 6.85	292.13 ± 9.69	0.718
PLT (10^9^/L)	781.18 ± 245.67	771.50 ± 135.17	0.902
MPV (fL)	10.57 ± 0.48	11.26 ± 0.98	0.225
Serum hormone level (*n* = 6/group)			
Testosterone (ng/mL)	0.30 ± 0.08	0.19 ± 0.01	0.105
Estradiol (pg/mL)	1.55 ± 0.18	0.63 ± 0.13	0.002
Proinflammatory factor			
IL-1β (pg/mg)	50.47 ± 4.66	64.2 ± 7.28	0.014
IL-6 (pg/mg)	674.36 ± 128.13	948.52 ± 115.44	0.008
TNF-α (pg/mg)	129.36 ± 11.8	158.83 ± 19.85	0.013
Oxidative stress parameters			
MDA (nmol/mg)	0.12 ± 0.02	0.15 ± 0.03	0.047
SOD (U/mg)	0.90 ± 0.07	0.49 ± 0.10	0.007
GSH-Px (U/μg)	34.75 ± 1.99	19.80 ± 1.47	<0.001
GSH (μg/mg)	0.3 ± 0.09	0.22 ± 0.03	0.104
CAT (nmol/min/mg)	0.4 ± 0.04	0.33 ± 0.05	0.045

### Ovariectomy effects on neurodegeneration and protein expression levels in the hippocampus of female chronic hypobaric-hypoxia-exposed rats

**FIGURE 12:**
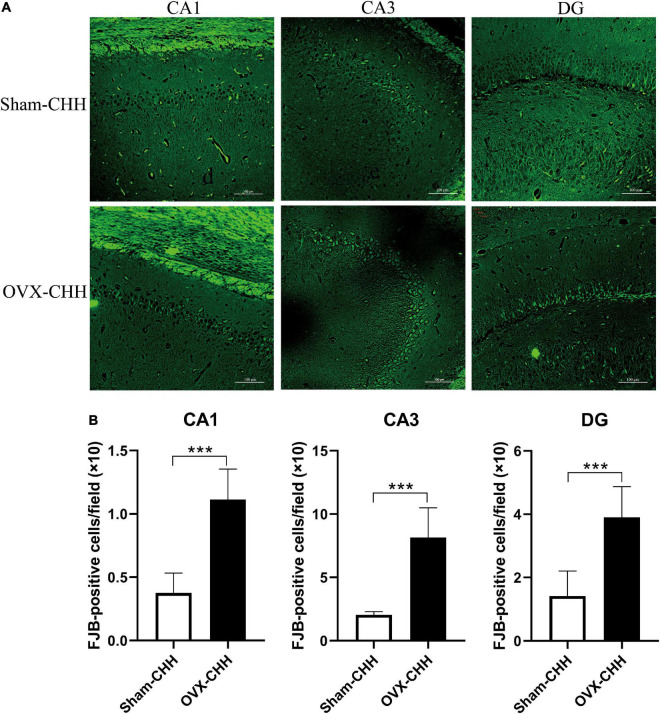
Chronic hypobaric-hypoxia exposure for 3 months, OVX showed more obvious hippocampal neuron death than intact females. **(A)** Representative immunofluorescence images of FJB staining in different hippocampal subregions (CA1, CA3, DG) (20×), Scale bar = 100 μm; **(B)** statistical analysis of FJB positive cells in different hippocampal subregions (CA1, CA3, DG). OVX, bilateral ovariectomy. Values are means ± SD for six animals/group. Compared with sham CHH, *p < 0.05, **p < 0.01, ***p < 0.001.

We hypothesized that OVX rats were more susceptible to CHH-induced hippocampal neuronal damage than sham-operated female rats due to ovarian hormone deficiency. Compared with the sham-CHH group, FJB positive cells in different hippocampal regions (CA1, CA3, and DG regions) of the OVX-CHH group increased significantly (*p* < 0.001) (Figures 12A,B). These results suggest that ovarian hormone deficiency possibly increased the risk of hippocampal neuronal degeneration under CHH exposure.

**FIGURE 13:**
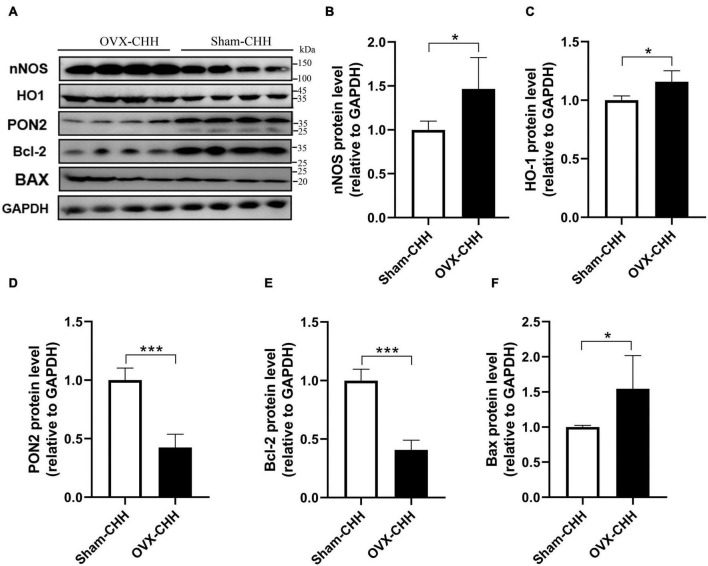
Effects of OVX on PON2, nNOS, HO-1, Bcl-2, and Bax protein expression in the hippocampus of female CHH rats. **(A)** Representative western blot images of nNOS, HO-1, PON2, Bcl-2, and Bax proteins, 30 μg/lane. **(B–F)** Quantitative analysis of relative optical density and comparison between groups. Values are means ± SD for four animals/group. Compared with sham CHH, *p < 0.05, **p < 0.01, ***p < 0.001.

The results of this study show that after ovarian hormone deficiency, the expression of nNOS, HO-1, and Bax protein increased whereas the expression of PON2 and Bcl-2 protein decreased in the hippocampus of female rats (Figures 13A–F).

## Discussion

Chronic hypobaric-hypoxia exposure can lead to hippocampal neuron damage and cognitive decline, which has been widely reported. However, there are few studies on the vulnerability of sex differences to hypoxia at high altitudes. In this experiment, we have made a major finding as regards such sex differences. In this study, in the animal model raised at a natural high altitude for 3 months, differences were found between the two sexes in hippocampal damage and cognitive deficits, which has not been reported previously. Compared with its respective control group, the male CHH group, rather than the female CHH group, showed a more significant decline in cognitive ability, more obvious neuronal injury, microgliosis, neuroinflammation, and oxidative stress in the hippocampus, and more severe decreased hippocampal integrity (such as neurogenesis and dendritic spine density), which are closely related to neurological deficits. Male rats seemed to be more vulnerable than female rats to hippocampal injury and cognitive impairment induced by CHH, possibly due to the low expression level of PON2 protein in males.

It has been reported that the hypobaric-hypoxia environment may be deleterious to male reproductive function, which is accompanied by a decrease in serum testosterone level (Liu et al., 2020). It has also been reported that hypobaric–hypoxia may affect female hormone levels, and the hormone levels of the menstrual cycle in high altitude areas are different from that at sea level (Escudero et al., 1996). In this study, the serum testosterone level of the male CHH group was lower than the male control group, and the serum estradiol level of the female CHH group was higher than the female control group, which was consistent with the previous reports (Liu et al., 2020; Pooja et al., 2020). Estrogen has antioxidant (Yazğan et al., 2016; Paris et al., 2021), anti-inflammatory (Fallarino et al., 2010; Siani et al., 2017), and neuroprotective effects (Cipolla et al., 2009; Robison et al., 2019), which was been widely reported. Although it has been reported in recent years that androgens also have anti-inflammatory (Mohamad et al., 2019; Babcock et al., 2022) and antioxidant (Ahlbom et al., 2001) effects, however, the protective effect of testosterone has a dichotomous effect. Once oxidative stress reaches a specific threshold, testosterone is no longer neuroprotective and low levels of testosterone may even aggravate the generation and damage of oxidative stress (Holmes et al., 2016). For example, men with sleep apnea and under elevated oxidative stress may be vulnerable to neurodegenerative pathophysiology (Snyder et al., 2018), which is probably related to sex and sex hormone differences in hippocampal formation and plasticity (Yagi and Galea, 2019). In turn, these sex differences may lead to greater susceptibility of males to hippocampal damage and cognitive impairment caused by hypoxia at high altitudes.

It has been reported that oxidative stress and decreased antioxidant capacity play an important mediating role in the neurophysiological disorders of memory impairment caused by hypobaric–hypoxia (Hota et al., 2007). In the current study, after 3 months of CHH exposure, the levels of proinflammatory cytokines (IL-6, IL-1β, and TNF-α) in the hippocampus of male animals increased, the content of MDA increased while the content of GSH and the activities of antioxidant enzymes (SOD, GSH-Px, and CAT) decreased. In contrast, in the hippocampus of female animals, only the level of IL-1β increased and the activity of CAT enzymes decreased. The results show that after CHH exposure, the levels of proinflammatory cytokines and oxidative stress in male rats were significantly higher than in female rats. Thus, these results may evidence that there are higher levels of oxidative stress and neuroinflammation in the hippocampus of male rats than in their female counterpart in the CHH-exposed environment.

Inflammation-induced by hypobaric-hypoxia may lead to brain injury and neuronal loss with long-lasting effects on the regional or global volume of the hippocampus. Moreover, inflammation also inhibits hippocampal neurogenesis (Monje et al., 2003). The reduced rGMV shown by our study reveals that regional atrophy happened mainly in the hippocampus head in male CHH rats after CHH exposure whereas no significant GMV change was observed in the female CHH and control rats. We have demonstrated that the bilateral hippocampus was one of the first affected regions identified by the imaging technique. The change in hippocampal GMV may be related to more neurodegeneration (Zhou et al., 2018) and severe reductions in dendritic arborization (Alexander et al., 2020) in the male CHH group, which are the known factors contributing to GMV reduction. Oxidative stress and neuroinflammation caused neurodegenerative changes characterized by neuronal loss and brain atrophy with corresponding memory impairment and cognitive deficits (Maiti et al., 2008).

Hippocampal neurons in male rats are more susceptible to CHH than in female rats. FJB staining shows that, after 3 months of CHH exposure, the neurodegeneration of male CHH-exposed rats was significantly higher than that of female CHH rats. The total dendritic length and dendritic spine density of hippocampal neurons in male CHH-exposed rats were significantly lower than in female CHH-exposed rats. This finding morphologically evidences that CHH exposure can induce more hippocampus-dependent spatial memory impairments in male CHH rats than in their female counterpart.

It has been accepted that learning, memory ability, and emotion regulation are related to neurogenesis in the subgranular zone of the DG region of the hippocampus (Ming and Song, 2011). BrdU^+^ cells can be expressed in neurons 21 days after BrdU incorporation (Kempermann et al., 2004). The results of our study show that CHH significantly reduced the number of BrdU^+^/NeuN^+^ labeled cells in the hippocampal DG of male rats whereas the number of BrdU^+^/NeuN^+^ labeled cells in the hippocampal DG of female rats was hardly affected. It has been confirmed by some studies (Koester-Hegmann et al., 2018; Chauhan et al., 2022) that CHH may also induce a decrease in DG neurogenesis in the hippocampus, however, with a sex difference. In addition, neuroinflammation can lead to abnormalities in adult brain neurogenesis. Prior studies have found that hypobaric-hypoxia stimulates microglia, astrocytes, and endothelial cells, and then produces neuroinflammation and neuronal degeneration in the hippocampal DG area (Chauhan et al., 2022). Microglia affects neurogenic niches by balancing pro-inflammatory and anti-inflammatory conditions, which can either promote or inhibit neurogenesis. The mechanism of CHH-induced reduction in neurogenesis in male rats may be ascribed to gliosis and neuroinflammation in male rats.

It has been reported that women are less likely to suffer from hypoxia-related syndrome than men probably because female sex hormones are involved in the mechanism of controlling women’s ventilatory response to hypoxia (Soliz et al., 2009). PON2 has been proved to have anti-inflammatory and antioxidant effects on the brain and other tissues (Aguirre-Vidal et al., 2020; Blackburn et al., 2022). The protective effect of PON2 on neurons and astrocytes is related to its ability to scavenge reactive oxygen species when exposed to oxidants. The sensitivity of PON2 to reactive oxygen species is associated with the relative content of PON2 (Giordano et al., 2011). A study has shown that the expression level of PON2 in women is significantly higher than in men in all tissues and all cell categories (Furlong et al., 2016). On the other hand, mediated by α-estrogen receptor activation, estradiol can increase the content of PON2 in astrocytes of the striatum in male and female mice in a time- and concentration-dependent manner (Giordano et al., 2013). Our study shows that the expression of PON2 protein in the hippocampus of female rats was significantly higher than male rats, which has further confirmed the sex difference in PON2 protein content in tissues in prior studies (Giordano et al., 2011). The relatively low expression level of PON2 in male rats’ hippocampus may indicate the potential lack of antioxidant defense ability.

After 3 months of CHH exposure, the hippocampal expression of PON2 protein in the male and female CHH groups was significantly lower than in the control group (*p* < 0.05). In both female and male rats, CHH exposure can reduce PON2 protein expression. Nevertheless, PON2 protein expression in the female CHH group was significantly higher than in the male CHH group (*P* < 0.05). Clinical and experimental evidence shows that oxidative stress and inflammation are the most important contributors to cognitive impairment and neuronal damage at high altitudes (Choudhary et al., 2017). Lower expression levels of PON2 can increase susceptibility to neuroinflammation and oxidative stress (Giordano et al., 2013; Ehsanifar et al., 2021). Based on the results of this study, we may comfortably suggest that male rats are more vulnerable to CHH-induced hippocampal neuronal damage than female rats, which may be related to the low expression level of PON2.

This study reveals that the expression of nNOS and HO-1 protein in rat hippocampus increased after CHH exposure. The expression of nNOS and HO-1 mRNA was also enhanced in male CHH-exposed rats, indicating that oxidative stress and immune system disorder were occurring in the CNS (central nervous system) of CHH-exposed rats. HO-1 is particularly sensitive to the induction of cytokines, oxidants, and other stressors (Schipper et al., 2009) and nNOS can induce an increase in nitric oxide production in the CNS (Czerniczyniec et al., 2015). Besides, the expression of nNOS is directly related to glutamate. Previous evidence suggests that CHH can alter the glutamatergic system in the brain (Hota et al., 2008). The hippocampus is rich in *N*-methyl-D-aspartic acid (NMDA) receptors (Ehsanifar et al., 2019). NMDA receptors, as has been reported, can regulate postsynaptic calcium influx, trigger a series of events, including NOS activation and excessive production of nitric oxide, and eventually lead to cell damage in the process of hypoxic injury (Huang et al., 2015). Elevated nitric oxide levels and increased nNOS expression may induce hypoxic neurodegeneration in the hippocampus (Maiti et al., 2007).

As an anti-apoptotic protein located in the outer membrane of mitochondria, Bcl-2’s overexpression in neurons can inhibit neuronal apoptosis by maintaining mitochondrial integrity (Wu et al., 2015). In rats, intermittent preconditioning with hypobaric-hypoxia can reduce hippocampal neuron apoptosis by locally up-regulating the Bcl-2 expression, thereby reducing the severity of brain damage after ischemia-reperfusion (Xing et al., 2008; Zhang et al., 2008). After CHH exposure, the expression of anti-apoptotic protein Bcl-2 in male rats’ hippocampus decreased. The pro-apoptotic gene Bax is reported to be significantly involved in mitochondrial-mediated neuronal apoptosis (Mesole et al., 2020). Given this consideration, we speculate that the decrease in Bcl-2 protein expression in CHH-exposed male rats may increase hippocampal neuronal apoptosis and aggravate the impairment of cognitive function.

NMDA is composed of different subunits such as NR1, NR2, and NR3, while NR1, NR2A, and NR2B are highly expressed in the hippocampus (Yu et al., 2020). NR2A or NR2B functions as an accessory subunit of NR1. In many learning and memory disorders, the change in the expression level of NMDA receptors containing NR2A and NR2B is considered a marker symbolic of abnormal long-term potentiation (Ieraci and Herrera, 2020; Yu et al., 2020). In the current study, the mRNA expression levels of NR2A and NR2B in the hippocampus were quantified by qRT-PCR. In female rats, although CHH exposure had a downward trend in the mRNA expression of NR2B in the hippocampus, it had hardly any effect on the mRNA expression of NR2A. We observed that the relative expressions of NR2A and NR2B were down-regulated in male CHH rats compared to the male control rats, which was similar to the findings of prior studies (Zhu et al., 2019). In light of this, we suggest that there are sex differences involved in the down-regulation of NR2A and NR2B subunits in CHH-exposed animals. In addition, the reduction in NR2A and NR2B receptor subunits may partly account for the memory impairment observed in the hippocampus of male CHH rats.

Compared with sham-CHH rats, OVX-CHH rats showed higher levels of oxidative stress and proinflammatory cytokines. Compared with sham-CHH rats, the expression of nNOS, HO-1, and Bax protein increased whereas the expression of PON2 and Bcl-2 protein decreased. The expression level of PON2 in women is usually significantly higher than that in men (Furlong et al., 2016), and the expression of PON2 in female mice is mostly significantly higher than that in male mice (by about three times in the brain) (Giordano et al., 2011), which may be ascribed to the enzyme estrogen balance (Levy et al., 2019). The current study found that the expression level of PON2 protein in the hippocampus of OVX-CHH male rats was lower than female rats in the sham-CHH group, indicating that the expression level of PON2 protein in the hippocampus decreased after bilateral ovariectomy. The reduced expression level of PON2 protein may be related to its weakened anti-inflammatory and neuroprotective effects, and thus can induce more oxidative stress, neuroinflammation, and neuronal death in CHH-exposed rats. Therefore, we may suggest that ovarian hormone deficiency is probably linked to a decrease in PON2 protein expression and that this decrease may be ascribed to the increased susceptibility to CHH-induced inflammation, oxidative stress, and degeneration of hippocampal neurons. These results suggest that ovarian hormones also play an important role in CHH-induced hippocampal damage, which may be related to PON2-mediated sex differences. These findings further evidence of sex differences in hippocampal damage and cognitive function induced by CHH exposure and lend support to the beneficial role of ovarian hormones in hippocampal neuronal degeneration induced by CHH exposure.

## Limitations

This study is not exempt from limitations of its own. First, the animals housed at high altitudes were transported back to standard conditions at plain to perform euthanasia rather than being euthanized at high altitudes. Moving the rats to standard conditions may likely affect the gene expression in these animals. Second, due to the lack of clearly defined specific substrates and standardized measurement techniques of these enzymes, the tissue distribution of PON2 could not be displayed by histopathological methods such as immunohistochemistry (Camps et al., 2009). Finally, we used the SD rat model of CHH exposure, which did not include dogs and non-human primates but should be used in future studies.

## Conclusion

In this study, male rats seem to be more vulnerable to the effect of hippocampal damage induced by chronic exposure to hypobaric hypoxia, which might be related to the low level of paraoxonase 2 (PON2) expression. Studying the sex differences in hippocampal damage and cognitive impairment caused by hypobaric-hypoxia may help explore the mechanism underlying the sex differences that hypobaric-hypoxia destroys hippocampal integrity and leads to cognitive impairment, which may be very important for the understanding of precision medicine.

## Data availability statement

The raw data supporting the conclusions of this article will be made available by the authors, without undue reservation.

## Ethics statement

The animal study was reviewed and approved by the Experimental Animal Ethics Committee of West China Hospital, Sichuan University, Chengdu, China. Written informed consent was obtained from the owners for the participation of their animals in this study.

## Author contributions

FG conceptualized the project. FG and DZ designed the study and drafted the manuscript. DZ, MZ, BH, YW, and LW contributed to the literature search, data collection and analysis, and data interpretation. FG critically revised the manuscript. All authors approved the final version of the manuscript.
